# Unveiling an Inverted Papilloma of the Bladder: A Case Series and Literature Review

**DOI:** 10.7759/cureus.95417

**Published:** 2025-10-25

**Authors:** Mohammad Ekhlasur Rahman, Muhammad Rakib Hasan, Mahabub Hassan, Kashif Waheed, Angelos Christofides, Bharati Tripathi

**Affiliations:** 1 Urology, Watford General Hospital, West Hertfordshire Teaching Hospitals NHS Trust, Watford, GBR; 2 Urology, Epsom and St Helier University Hospitals NHS Trust, Sutton, GBR; 3 Histopathology, Watford General Hospital, West Hertfordshire Teaching Hospitals NHS Trust, Watford, GBR

**Keywords:** bladder inverted papilloma, hematuria, transurethral resection, urinary bladder neoplasms, urothelial tumors

## Abstract

Bladder inverted papilloma (BIP) is a rare, benign urothelial lesion that can mimic urothelial carcinoma endoscopically but is characterized histologically by an endophytic growth of anastomosing urothelial cords without muscular invasion. We report a single-center case series of four patients managed with complete transurethral resection between January 2022 and August 2025, with the inclusion criterion being a histopathological diagnosis: a 28-year-old man with a trigonal lesion complicated by bladder perforation that healed uneventfully, a 72-year-old man in whom a small peri-orificial lesion was resected during urgent ureteric stenting and subsequently followed on a low-risk bladder cancer pathway with no recurrence at three years, an 84-year-old man with a large polypoid lesion arising near the bladder neck that was completely excised, and a 72-year-old woman with a pedunculated lateral wall mass treated by resection and single-dose intravesical mitomycin, remaining disease-free at one year. Synthesizing contemporary evidence and our experience, we highlight that accurate histopathological confirmation, complete endoscopic excision, and multidisciplinary discussion are key to management; routine intensive cystoscopic surveillance appears unnecessary for unequivocal bladder BIP, whereas selective follow-up is reasonable when pathology raises a differential of low-grade urothelial neoplasm with inverted growth or when lesions arise outside the bladder, where malignant association is higher. These findings support a pragmatic, risk-adapted follow-up strategy after complete resection.

## Introduction

Bladder inverted papilloma (BIP) is an uncommon urothelial lesion (1-2% of bladder tumors) defined by an endophytic proliferation of cytologically bland urothelium forming anastomosing cords and trabeculae within the lamina propria, without true muscular invasion [1,2].

It typically affects older men and presents with painless hematuria or irritative voiding symptoms; cystoscopically, lesions often arise at the bladder neck or trigone and can mimic papillary urothelial carcinoma [1]. Although historically regarded as benign with low recurrence after complete transurethral resection, the literature is conflicted regarding malignant association: some series advocate routine surveillance because a minority develop synchronous or metachronous urothelial carcinoma, whereas others, when strict histologic criteria are met and resection is complete, find no progression and question prolonged surveillance [1-4].

Molecular data increasingly support biologic distinctness: targeted sequencing found *FGFR3* mutations in a subset of BIP and rare/absent TP53 changes [2], while contemporary next-generation sequencing (NGS) shows predominant RAS pathway alterations (HRAS/KRAS) in classic BIP and contrasts these with FGFR3/TERT-enriched inverted-growth carcinomas, aligning with the broader paradigm that FGFR3- and TP53-driven urothelial tumors represent largely separate pathways [5-7]. This case series highlights diverse presentations and management decisions in practice, and proposes pragmatic follow-up aligning with evidence that balances vigilance against over-surveillance in patients with completely resected, classic BIP.

## Case presentation

Case 1

**Figure 1:**
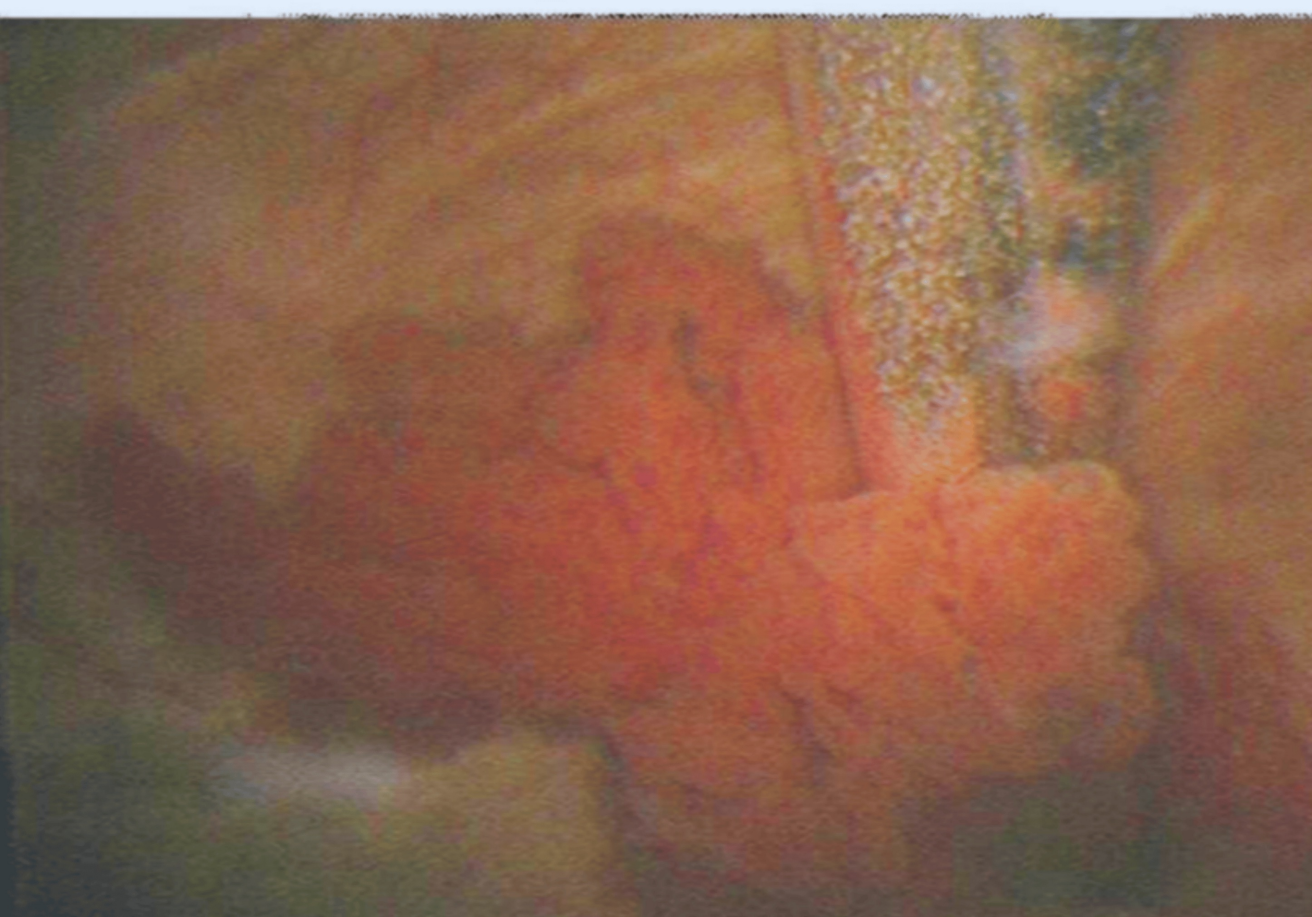
Flexible cystoscopy showing a papillary lesion arising from the trigonal/posterior wall region (Case 1) Endoscopic view of the bladder demonstrating a papillary, exophytic mucosal lesion. The lesion projects into the lumen with a lobulated surface; no active bleeding is seen in this frame. Instrument sheath is visible adjacent to the mass, which was subsequently resected at transurethral resection of bladder tumor (TURBT).

**Figure 2:**
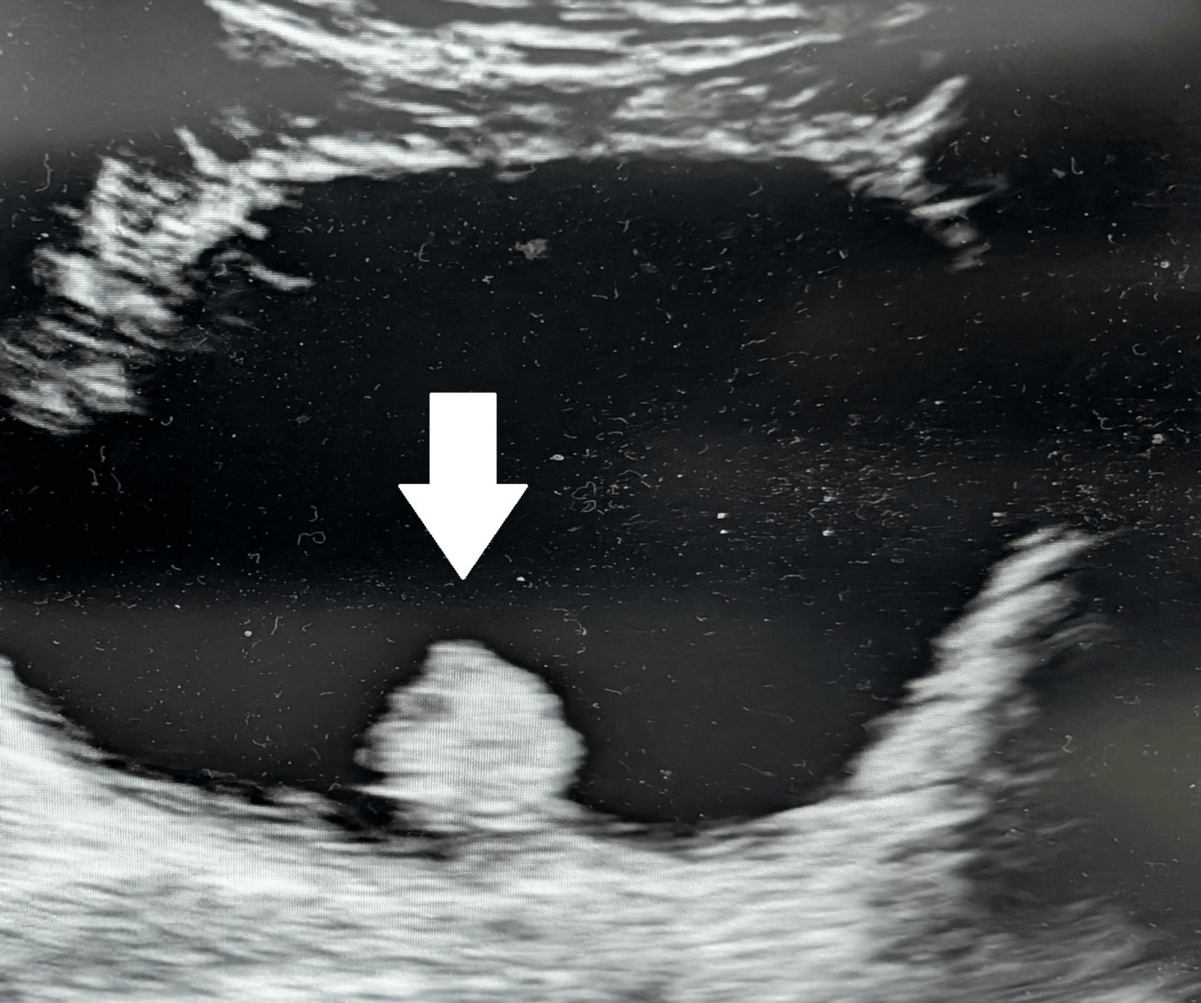
Ultrasound reveals a solitary 3-cm intravesical mass arising from the posterior wall in the trigonal region (Case 1) Bladder ultrasound demonstrates a polypoid, echogenic intraluminal lesion projecting from the posterior wall in the trigonal region (arrow). The lesion outlines smoothly toward the bladder lumen without acoustic shadowing, in keeping with an intravesical mass (~3 cm) later confirmed endoscopically.

A 28-year-old man presented with recurrent episodes of gross hematuria. Ultrasound revealed a solitary 3-cm intravesical mass arising from the posterior wall in the trigonal region. Flexible cystoscopy confirmed a papillary-appearing lesion centered near the trigone (Figures 1, 2).

**Figure 3:**
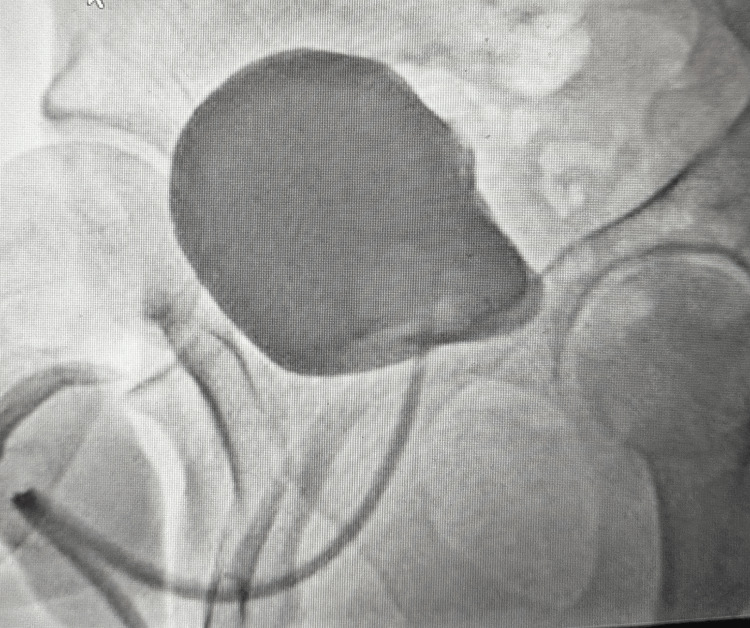
Cystography performed prior to catheter removal demonstrated complete healing (Case 1) Fluoroscopic cystogram performed prior to catheter removal shows uniform opacification of the bladder with a smooth, continuous serosal outline and no contrast extravasation into the perivesical space, consistent with complete healing after bladder perforation repair. A catheter is in situ for contrast instillation.

The patient underwent complete transurethral resection of bladder tumor (TURBT). A single immediate postoperative instillation of intravesical mitomycin was administered. Within the early postoperative period, he re-presented with hematuria and was returned to the operating room for bladder irrigation; an intraoperative bladder wall perforation was identified and repaired. Cystography performed before catheter removal demonstrated complete healing (Figure 3).

**Figure 4:**
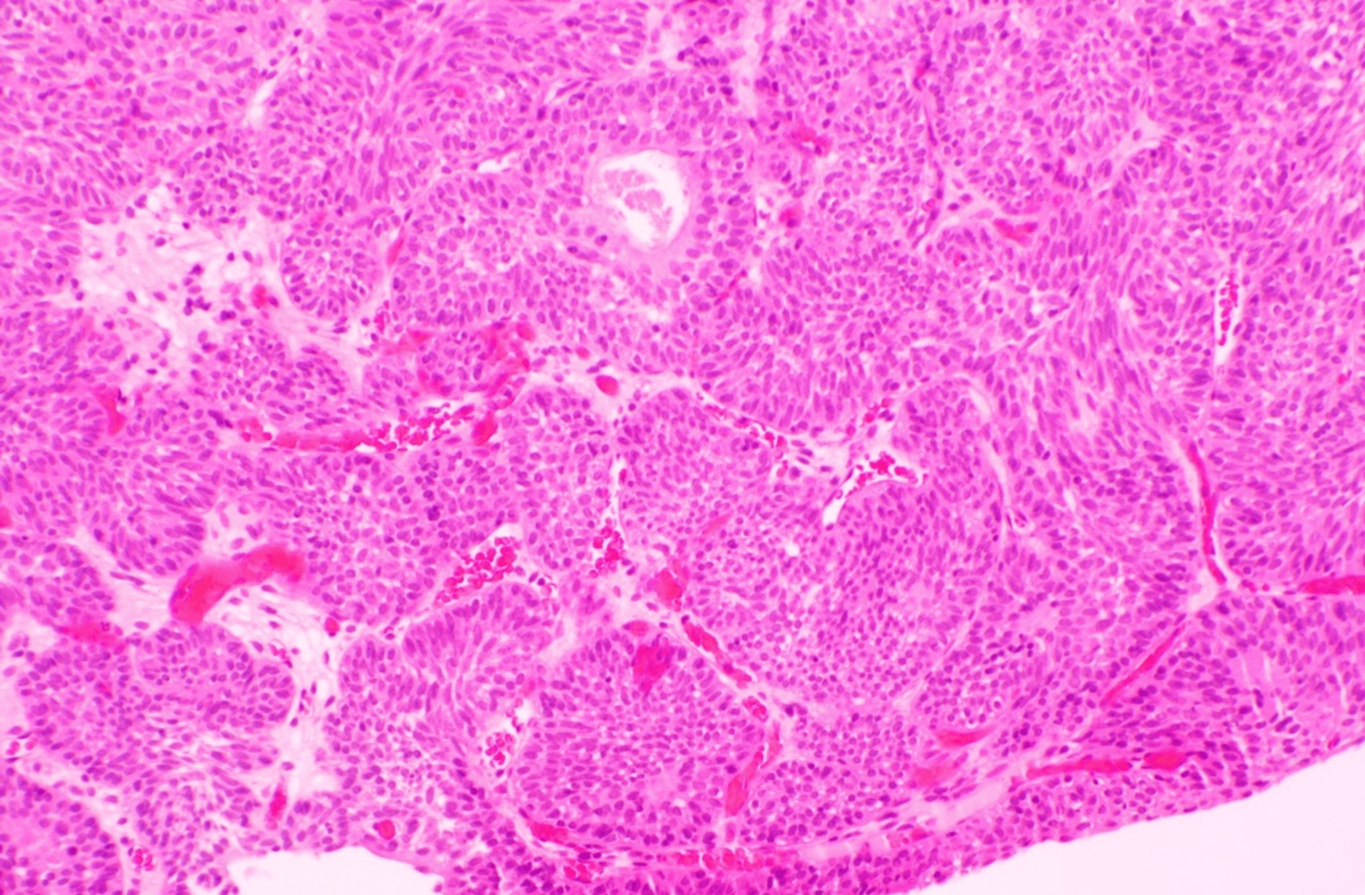
Benign inverted (endophytic) urothelial proliferation consistent with inverted papilloma—no cytologic atypia or invasion (Case 1) Sections show broad, smooth-contoured nests and anastomosing trabeculae extending into the lamina propria. The lining cells are cytologically bland with uniform nuclei, preserved maturation, and absent/rare mitoses. There is no stromal desmoplasia, no muscularis propria invasion, and no papillary fibrovascular cores, supporting a diagnosis of inverted papilloma rather than low-grade urothelial carcinoma with an inverted growth pattern.

Histopathological examination showed an inverted (endophytic) urothelial proliferation without cytologic atypia or invasion, in keeping with inverted papilloma and without malignant features. The patient made an uneventful recovery thereafter and was discharged with routine urology follow-up (Figure 4).

Case 2

A 72-year-old man presented to the accident and emergency (A&E) department with acute pain consistent with renal colic. Imaging confirmed a 10 mm calculus at the left pelvi-ureteric junction (PUJ). He underwent urgent ureteric stent insertion. During cystoscopy at the time of stent placement, a small papillary mucosal lesion was noted just lateral to the left ureteric orifice and was resected in the same sitting.

**Figure 5:**
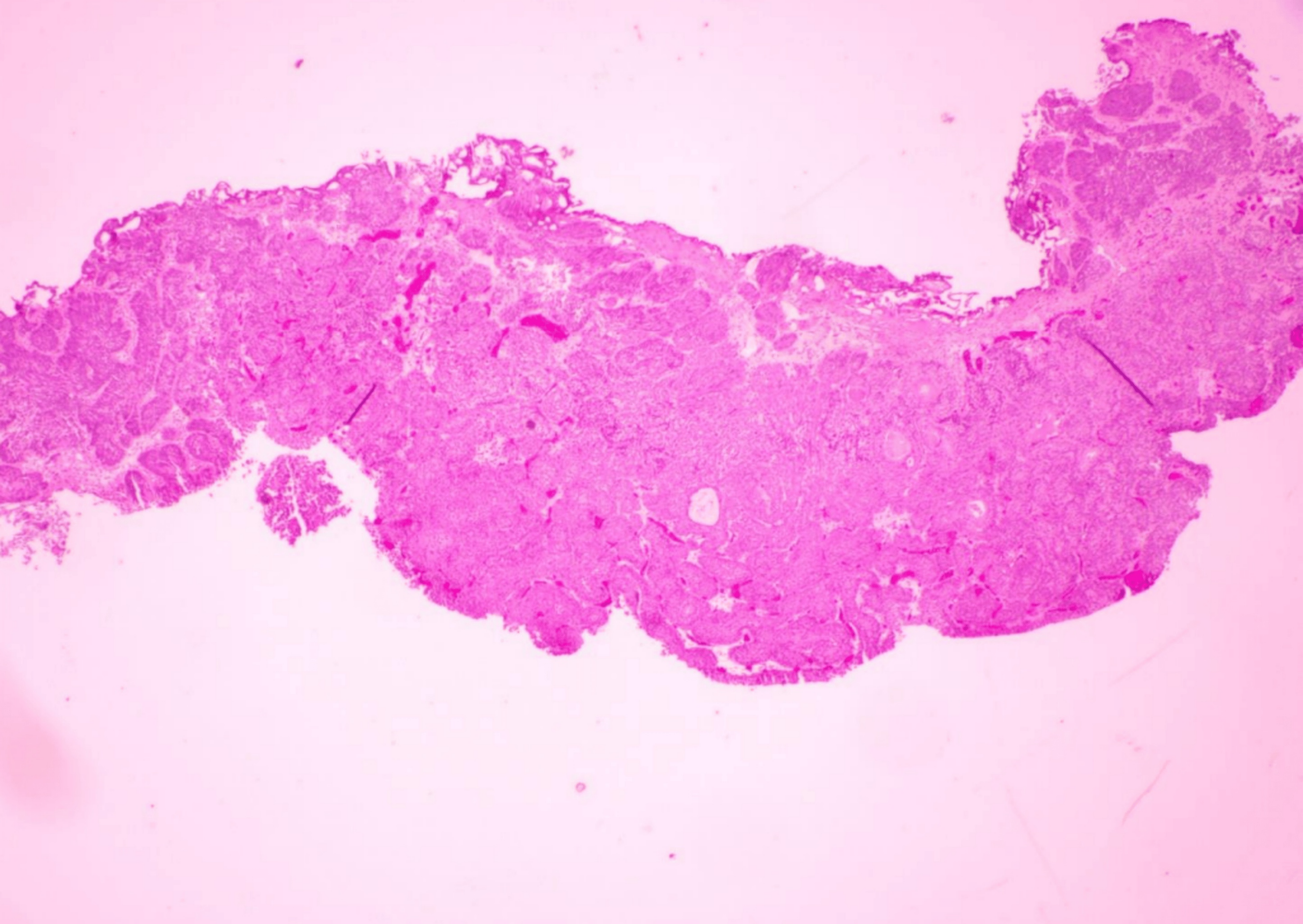
Panoramic H&E section showing inverted growth pattern of urothelial lesion (Case 2) Histopathology shows a predominantly endophytic/inverted architecture with broad, smooth-contoured nests and anastomosing trabeculae of urothelium extending into the lamina propria. The nests maintain maturation toward the center, and there is no stromal desmoplasia, papillary fibrovascular cores, or infiltrative/irregular edges, supporting an inverted papillomatous growth pattern rather than a papillary or invasive urothelial neoplasm

Histopathological examination described occasional small papillary surface structures. Overall, the features favored an inverted papilloma; however, a low-grade urothelial neoplasm with an inverted growth pattern was considered in the differential. The case was reviewed at the urology multidisciplinary team (MDT) meeting, where the consensus diagnosis was inverted papilloma (Figure 5).

The patient was subsequently enrolled in a low-risk non-muscle-invasive bladder cancer surveillance pathway according to institutional protocol. Over 36 months of follow-up, there has been no evidence of recurrence on surveillance evaluations.

Case 3 

**Figure 6:**
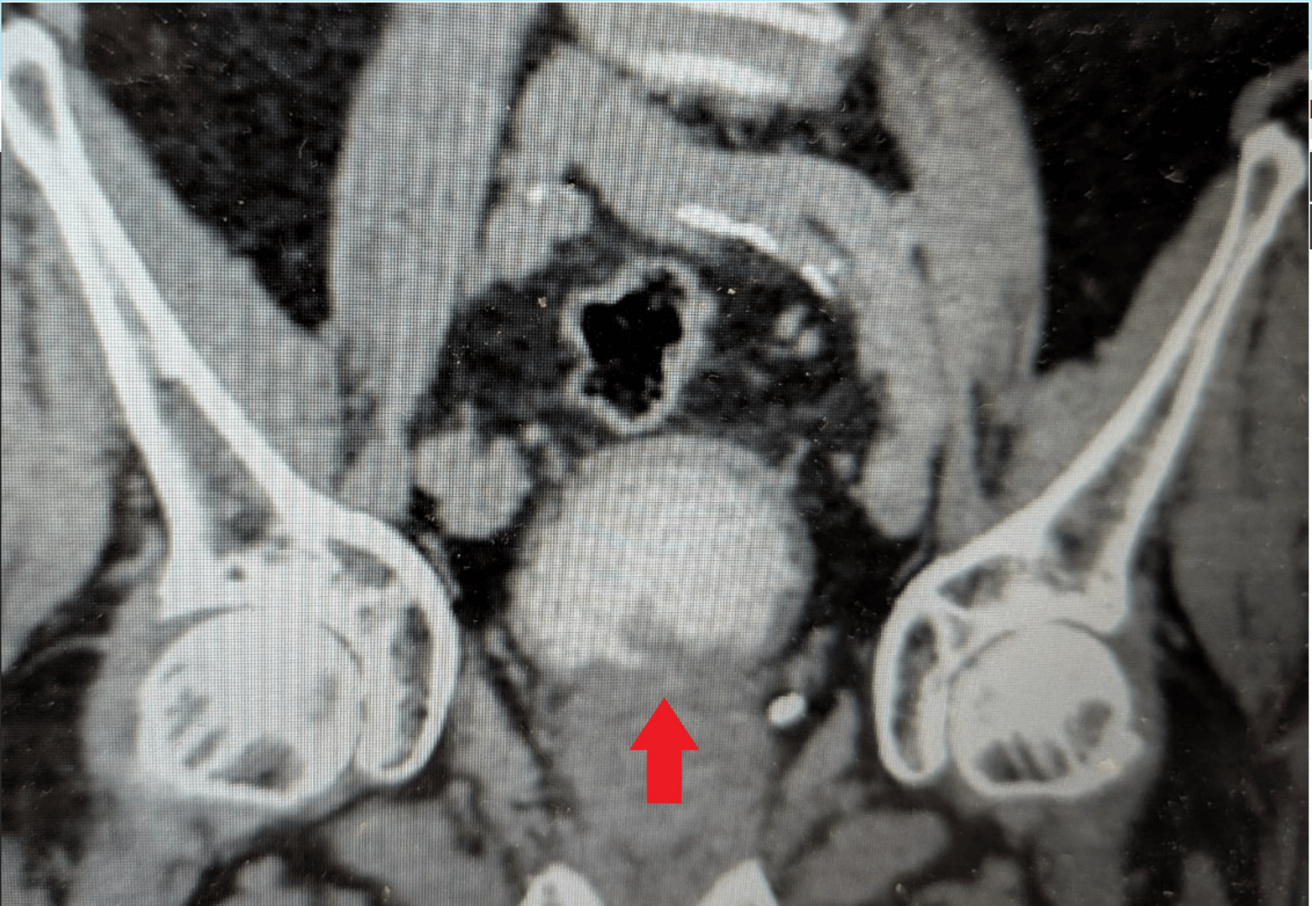
CT urogram showing small bladder filling defect (Case 3) A well-circumscribed intraluminal filling defect is seen within the urinary bladder (red arrow), compatible with a polypoid lesion. No upstream hydroureteronephrosis or additional pelvic abnormalities are evident on this slice, concordant with a solitary bladder lesion later confirmed endoscopically.

An 84-year-old man was referred via the urgent suspected cancer (two-week wait) pathway with hematuria. Flexible cystoscopy identified a solitary 5 cm intravesical lesion along the lateral bladder wall. Preoperative contrast-enhanced CT demonstrated a normal upper urinary tract and confirmed a single bladder lesion without additional abnormalities (Figure 6).

**Figure 7:**
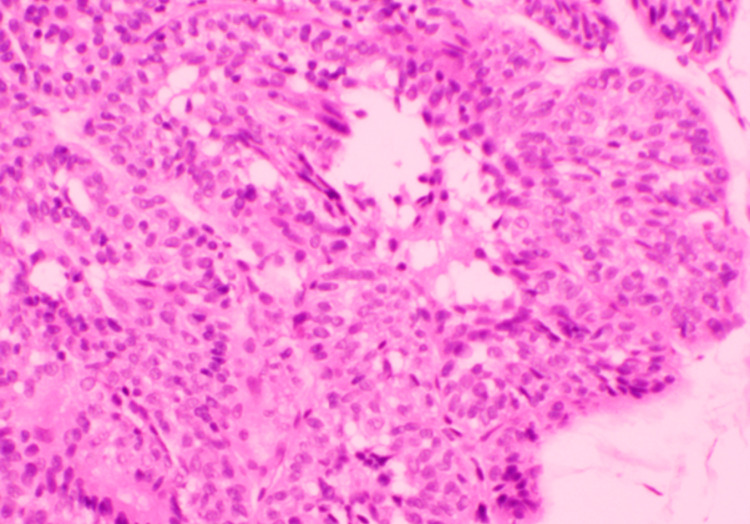
Pathological examination demonstrated an endophytic (inverted) pattern of urothelial growth (Case 3) High-power H&E shows endophytic/inverted growth with broad nests and anastomosing trabeculae of urothelium extending into the lamina propria. The lining cells are cytologically bland (uniform nuclei, preserved maturation, inconspicuous mitoses) with smooth, non-infiltrative borders. Papillary fibrovascular cores, stromal desmoplasia, necrosis, and invasion are absent, confirming a diagnosis of inverted papilloma.

Under general anesthesia, rigid cystoscopy showed a polypoid, pedunculated mass arising from the bladder neck with a stalk extending from the prostatic urethra and projecting into the bladder. Complete endoscopic resection was performed, and the specimen was submitted for histopathological evaluation. Histology was diagnostic of inverted papilloma (Figure 7). The patient had an uncomplicated postoperative course and was discharged with routine urological follow-up according to local protocol.

Case 4

**Figure 8:**
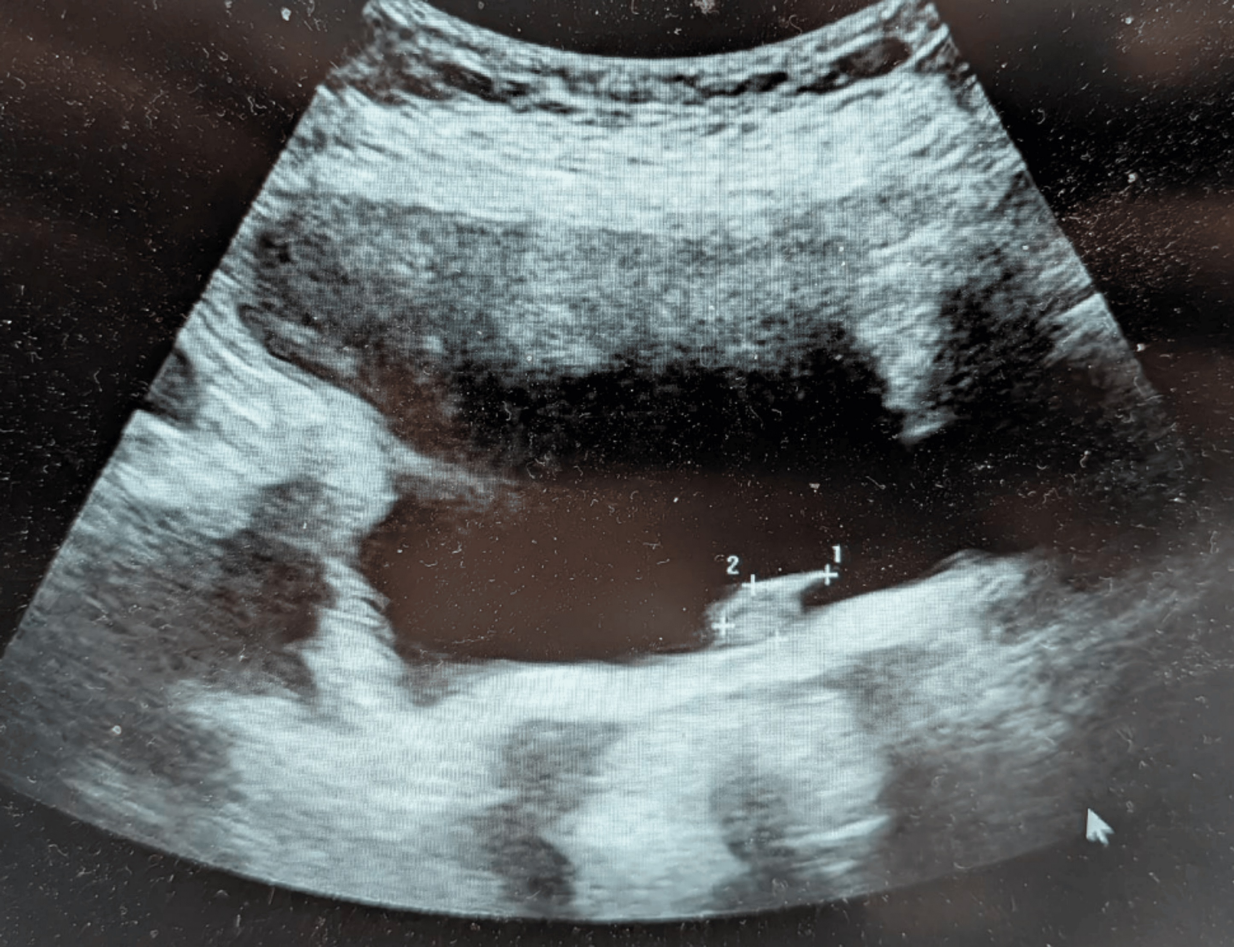
Ultrasound bladder showing bladder lesion (Case 4)

A 72-year-old postmenopausal woman presented with vaginal bleeding. A transvaginal ultrasound performed during the gynecologic evaluation incidentally identified an intravesical lesion. No concomitant urinary symptoms were documented. Diagnostic cystoscopy demonstrated a solitary lesion on the left lateral bladder wall. Endoscopically, the mass appeared pedunculated rather than papillary (Figure 8).

**Figure 9:**
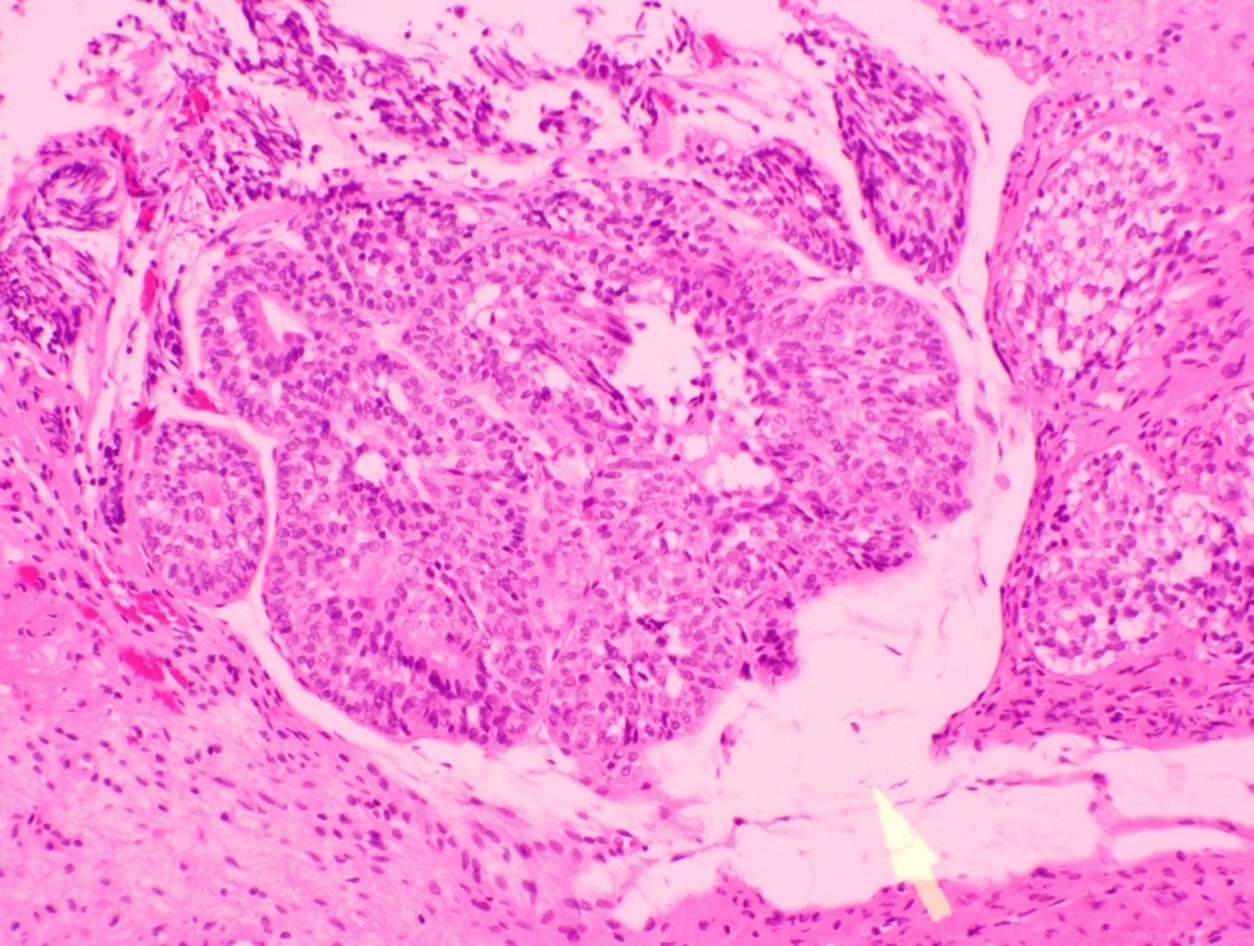
Histology of the resected specimen confirmed an inverted urothelial papilloma (Case 4) TURBT specimen shows broad, smooth-contoured nests and anastomosing trabeculae of urothelium growing inverted into the lamina propria. Tumor cells are cytologically bland with preserved maturation and inconspicuous mitoses. There is no stromal desmoplasia, necrosis, papillary fibrovascular cores, or invasive pattern, consistent with an inverted urothelial papilloma. No malignant features are present in the submitted material; the resection base shows cautery change. TURBT: transurethral resection of bladder tumor

The lesion was completely resected transurethrally under general anesthesia. A single immediate postoperative instillation of intravesical mitomycin C was administered as per institutional protocol. Histology of the resected specimen confirmed an inverted urothelial papilloma with endophytic architecture. No malignant features were reported in the submitted material (Figure 9).

The patient's postoperative recovery was entirely uneventful, with no procedure-related complications or readmissions. She was subsequently placed on routine surveillance for a full year, attending scheduled follow-up assessments throughout this period. The bladder remained clear with no evidence of recurrence, and, after 12 months of negative evaluations, she was discharged from urologic care.

## Discussion

This case series highlights the clinical variability of BIP. Classically, an uncommon benign tumor (1-2% of bladder neoplasms) is seen in older men [4,6-9]. Our patients spanned a broad spectrum. Although the literature reports a mean age of ≈60 (range 20-88) with a roughly 6:1 male predominance [10-12], we encountered a 28-year-old man and a 72-year-old woman, echoing recent reports of atypical presentations [4,6].

Symptoms in our series ranged from classic gross hematuria (in two patients) to incidental findings during evaluation for unrelated issues (two patients). BIP often causes painless hematuria but can be asymptomatic or cause irritative voiding [6,9]. Cystoscopically, BIP typically appears as a smooth, pedunculated, or papillary lesion [1,2,6,7]. In all our cases, histology showed the characteristic inverted (endophytic) growth of bland urothelium, clearly distinguishing it from malignancy.

All lesions were managed with complete transurethral resection. Two patients also received intraoperative intravesical mitomycin (reflecting initial concern for a papillary tumor). One patient suffered a bladder perforation requiring repair during resection, reminding us that procedural complications can occur even when treating benign-appearing lesions. At follow-up (up to three years), no patient has had tumor recurrence or new urothelial carcinoma. This excellent outcome mirrors published experience: for example, in a series of 20 patients with BIP (median 30-month follow-up), no recurrences were observed [1].

The question of surveillance intensity after BIP resection is debated. Although BIP itself is benign, occasional urothelial carcinomas have been reported before, during, or after BIP [7,13]. Accordingly, some experts recommend routine flexible cystoscopic monitoring [4,7]. Others argue that after a solitary BIP is fully excised with no concurrent carcinoma, intensive long-term follow-up may not be needed [14]. We therefore adopted a pragmatic approach: one patient entered a low-risk bladder surveillance protocol, while the others underwent periodic cystoscopy per standard low-risk guidelines. This balanced strategy aims to catch the rare concerning case without overburdening patients with excessive investigations. 

These cases underscore that BIP can present in diverse clinical contexts. Awareness of this spectrum in patient age, sex, presenting symptoms, and lesion appearance is important for accurate diagnosis. Given the uniformly benign post-resection course we observed (and similar reports in the literature), management should focus on thorough endoscopic resection. Follow-up can then be tailored to individual risk, with routine cystoscopic checks but without excessive intensity. Future pooled analyses may further refine optimal surveillance strategies.

## Conclusions

Inverted papilloma is a benign urothelial tumour with very low recurrence and good prognosis. It is not considered a risk factor for transitional cell carcinoma (TCC), especially when in the bladder. Complete transurethral resection is usually sufficient, and intensive TCC-style follow-up is not required.

## References

[1] Sweeney MK, Rais-Bahrami S, Gordetsky J (2017). Inverted urothelial papilloma: a review of diagnostic pitfalls and clinical management. Can Urol Assoc J.

[2] Brown AL, Cohen RJ (2011). Inverted papilloma of the urinary tract. BJU Int.

[3] Sung MT, Maclennan GT, Lopez-Beltran A, Montironi R, Cheng L (2006). Natural history of urothelial inverted papilloma. Cancer.

[4] Picozzi S, Casellato S, Bozzini G (2013). Inverted papilloma of the bladder: a review and an analysis of the recent literature of 365 patients. Urol Oncol.

[5] Lott S, Wang M, Zhang S (2009). FGFR3 and TP53 mutation analysis in inverted urothelial papilloma: incidence and etiological considerations. Mod Pathol.

[6] Kanakakis I, Giannoulis C, Tzelves L, Vlachodimitropoulos D, Berdempes M (2025). Inverted urothelial papilloma in a young woman: a case report and review of diagnostic advances. Cureus.

[7] Ouskri S, Kadouri Y, Lakssir J, Boualaoui I, El Sayegh H, Nouini Y (2025). Inverted papilloma of the bladder: a very rare benign lesion with malignant implications - case report and comprehensive literature review. Int J Surg Case Rep.

[8] Takeuchi M, Sasaguri K, Naiki T (2015). MRI findings of inverted urothelial papilloma of the bladder. AJR Am J Roentgenol.

[9] Limaiem F, Pandey J, Leslie SW (2023). Inverted urothelial papilloma. StatPearls [Internet].

[10] Patel P, Reikie BA, Maxwell JP, Yilmaz A, Gotto GT, Trpkov K (2013). Long-term clinical outcome of inverted urothelial papilloma including cases with focal papillary pattern: is continuous surveillance necessary?. Urology.

[11] Cheng CW, Chan LW, Chan CK (2005). Is surveillance necessary for inverted papilloma in the urinary bladder and urethra?. ANZ J Surg.

[12] Kilciler M, Bedir S, Erdemir F, Ors O, Kibar Y, Dayanc M (2008). Evaluation of urinary inverted papillomas: a report of 13 cases and literature review. Kaohsiung J Med Sci.

[13] Guo A, Liu A, Teng X (2016). The pathology of urinary bladder lesions with an inverted growth pattern. Chin J Cancer Res.

[14] Ho H, Chen YD, Tan PH, Wang M, Lau WK, Cheng C (2006). Inverted papilloma of urinary bladder: is long-term cystoscopic surveillance needed? A single center's experience. Urology.

